# The efficacy and safety of vincristine, irinotecan and anlotinib in Epithelioid Sarcoma

**DOI:** 10.1186/s12885-024-11921-7

**Published:** 2024-02-03

**Authors:** Lu Xie, Xin Sun, Jie Xu, Xin Liang, Kuisheng Liu, Kunkun Sun, Rongli Yang, Xiaodong Tang, Wei Guo

**Affiliations:** 1 Musculoskeletal Tumor Center, Peking University People’s Hospital, No. 11 Xizhimen South Street, Xicheng District, Beijing, P. R. China; 2 Pathology Department, Peking University People’s Hospital, No. 11 Xizhimen South Street, Xicheng District, Beijing, P. R. China

**Keywords:** Epithelioid sarcoma, Systemic treatment, Objective response, Toxicity

## Abstract

**Background:**

Epithelioid sarcoma is a rare soft tissue sarcoma characterized by SMARCB1/INI1 deficiency. Much attention has been paid to the selective EZH2 inhibitor tazemetostat, where other systemic treatments are generally ignored. To explore alternative treatment options, we studied the effects of irinotecan-based chemotherapy in a series of epithelioid sarcoma patients.

**Methods:**

We retrospectively reviewed data from patients with metastatic or unresectable epithelioid sarcoma at the Peking University People’s Hospital treated with irinotecan (50 mg/m^2^/d d_1-5_ Q3W) in combination with Anlotinib (12 mg Qd, 2 weeks on and 1 week off) from July 2015 to November 2021.

**Results:**

A total of 54 courses were administered. With a median follow up of 21.2 months (95% CI, 12.2, 68.1), the 5-year overall survival rate was 83.3%. Five of eight (62.5%) patients presented with unresectable localized lesions, including local tumor thrombosis and lymphatic metastasis. The other patients had unresectable pulmonary metastases. Six of eight (75%) patients had progressed following two lines of systemic therapy. The objective response rate reached 37.5% (three of eight patients) while stabilized disease was observed in 62.5% (five of eight) of patients. No patient had progressed at initial evaluation. At the last follow up, two patients were still using the combination and three patients had ceased the therapy due to toxicities such as diarrhea, nausea, and emesis. One patient changed to tazemetostat for maintenance and one patient stopped treatment due to coronavirus disease 2019 (COVID-19). Another patient stopped therapy as residual lesions had been radiated.

**Conclusions:**

The combination of irinotecan and Anlotinib as a salvage regimen may be considered another effective treatment option for refractory epithelioid sarcoma.

**Trial registration:**

This study was approved in the Medical Ethics Committee of Peking University People’s Hospital on October 28, 2022 (No.: 2022PHD015-002). The study was registered in Clinicaltrials.gov with identifier no. NCT05656222.

## Introduction

Epithelioid sarcoma (ES) is a rare subtype of soft-tissue sarcoma of uncertain cellular origin that is characterized by failed expression of the SMARCB1/INI1 tumor-suppressor gene. This leads to the unopposed, constitutive, oncogenic activation of EZH2, an enzyme that trimethylates lysine 27 of histone H3 (H3K27me3) [1]. The rarity of ES in both pediatric and adult populations limits the available data on its natural history and treatment. SL Spunt, N Francotte, GL De Salvo, Y-Y Chi, I Zanetti, A Hayes–Jordan, SC Kao, D Orbach, B Brennan, AR Weiss, et al. [2] reviewed ES patients < 30 years old enrolled in two international prospective clinical trials and concluded that the estimated 5-year survival was 86.4%, 63.5%, and 0%, for low-, intermediate-, and high-risk patients, respectively. In addition, partial response was observed in 11/22 patients receiving neoadjuvant therapy (50%). Recently, M Gounder, P Schöffski, RL Jones, M Agulnik, GM Cote, VM Villalobos, S Attia, R Chugh, TW-W Chen, T Jahan, et al. [3] completed an international, open-label, phase-2 basket study and found that for locally advanced or metastatic ES, tazemetostat induced an objective response rate (ORR) of 15% (95% CI 7–26%). The median duration of response (DOR) was not reached at a median follow-up of 13.8 months (IQR 7.8–19.0). This suggests that in metastatic or advanced ES not eligible for complete resection, this epigenetic modifier is a good candidate for maintenance.

The backbone of standard treatment for localized ES is wide surgical excision, with radiation therapy utilized in cases at higher risk of local relapse [1]. However, systemic therapy should not be restricted to doxorubicin or tazemetostat alone in metastatic settings [1]. In some cases, alternative chemotherapy drugs or drug combinations may induce a more favorable response, which may be helpful in shrinking the tumor and improving surgical options. Patients may only cease taking medications when all of the residual tumors are eradicated via local treatment. In 2012, the earliest study on chemotherapy in epithelioid sarcoma had reported that systemic chemotherapy provided satisfactory palliation with a median progression-free survival (PFS) of 29 weeks (95% CI 23–35) [4]. Anthracycline-based regimens (usually doxorubicin in combination with ifosfamide) are commonly used as a first-line treatment. This treatment has been associated with an overall response rate ranging from 0 to 43% and a median progression-free survival (PFS) of three to eight months [5–8]. Gemcitabine, both as monotherapy or in combination with docetaxel, has a response rate in the range of 27–58% and a median PFS of 4–8 months [9, 10]. Signs of drug activity in a few cases have also been reported with pazopanib [11, 12] and dasatinib [13]. However, there is limited data on the activity of immunotherapy in ES, with one report of a response ascribed to pembrolizumab [14].

Irinotecan is a camptothecin analogue that was initially approved by the US Food and Drug Administration for the treatment of colorectal cancer in 1996 using a single, high-dose schedule [15]. This drug has taken on growing importance in the treatment of pediatric sarcomas, such as Ewing sarcoma and rhabdomyosarcoma, using a protracted administration schedule [16–18]. SN-38 is the active metabolite of irinotecan, and it mediates cytotoxicity by stabilizing the DNA topoisomerase I complex created during replication, preventing re-ligation of DNA, and restricting the activity of the topoisomerase I enzyme. In a preclinical study, we showed its efficacy in multiple soft tissue sarcomas [19–21], thus we tested this agent in combination with the anti-angiogenesis tyrosine kinase inhibitor (TKI) Anlotinib in refractory ES via off-label use.

In the current study, we retrospectively reviewed the records of patients treated with irinotecan, vincristine, and Anlotinib (VIA) with the following purposes: (1) to establish whether the VIA regimen is effective in metastatic or unresectable ES, including ORR and DOR and (2) to examine the tolerability of the VIA regimen in heavily treated patients with refractory metastatic or unresectable ES.

## Methods

### Eligibility

Data for the present analysis were retrospectively collected through the electronic medical record database of ES patients treated at Peking University People’s Hospital between July 2015–November 2021. Written informed consent was waived by the Medical Ethics Committee of Peking University People’s Hospital. The study met the requirements of the declaration of Helsinki and was carried out in accordance with the regulations of the local ethical committee.

Patients were selected according to the following criteria: (1) Grade 2 or 3 ES confirmed histologically using the American Joint Committee on Cancer (AJCC) system [22]; (2) patients presented with measurable lesions using the Response Evaluation Criteria In Solid Tumors (RECIST1.1) [23] and were not amenable to surgical resection or radiotherapy; (3) primary or secondary metastatic disease; (4) received more than two courses of the VIA regimen; (5) no concurrent treatment was given while on the VIA regimen; and (6) follow-up information and evaluation after chemotherapy were available.

### Regimen

Treatment typically consisted of a 90-minute intravenous infusion of irinotecan at a dose of 50 mg/m^2^/d for 5 days every 3 weeks, vincristine given at a dose of 1.4 mg/m^2^ (maximum 2 mg) on days 1 and 8, and oral administration of Anlotinib once daily on days 1–14 within a 21-day cycle. This regimen had been previously tested in our IB trial for dose climbing [19, 20]. Routine radiographic evaluation was carried out once every 6 weeks, and the follow-up interval for patients ceasing treatment was generally every two months. Antiemetics were given three days before and after chemotherapy. Prophylactic therapies for diarrhea, such as antibiotics, probiotics, activated charcoal, or alkalinization, were routinely administrated due to our previous experience with this combination in Ewing sarcoma patients [19, 20]. Myeloid growth factor support between cycles was given when hematologic toxicity was observed.

### Pathological evaluation and study parameters

Pathological reviews and SMARCB1 protein expression assays were performed in the Pathology Department of the Peking University People’s Hospital. Next-generation sequencing (NGS) was carried out by the Berry Oncology Corporation (Fuzhou, China). In addition to the data available in the database, surgical, reference pathology, radiology, and radiotherapy reports were studied by the first author. The regular protocol for patients with refractory sarcoma in our hospital consisted of baseline assessment via chest computed tomography (CT, with each layer ≤ 5 mm) and a bone scan or [^18^F]2-fluoro-2-deoxy-D-glucose-positron emission tomography (FDG-PET). If lesions other than lung metastases were identified, CT and/or magnetic resonance imaging (MRI) of those lesions was required. Clinical evaluation was assessed using the RECIST 1.1 criteria. PFS was analyzed using the Kaplan–Meier Method [24]. All of the statistical analyses were performed using SPSS 19.0 software (SPSS Inc., Chicago, IL, USA).

## Results

### Patient characteristics and demography

Between July 2015 and November 2021, a total of eight patients and 54 treatment courses were identified. The characteristics of the patients included in this study are summarized in Table 1. At initial diagnosis, the median age of all of the eligible patients was 36.0 years (range: 20.0–69.0 years). A female predominance (62.5%, 5/8) was noted in this cohort of patients. Among these patients, seven of eight (87.5%) had a primary lesion in the extremities while only one patient had multiple bone metastatic lesions with the primary lesion site unknown. No patient had primary lesions in the axial skeleton. The Eastern Cooperative Oncology Group (ECOG) score [25] was relatively low, with only one of eight patients (12.5%) having a score of more than two. Before receiving the combination therapy, two patients were treatment naïve, one had even progressed on first-line chemotherapy (doxorubicin and ifosfamide), four patients had progressed on two lines of systemic treatment (Table 2) consisting of various anti-angiogenesis TKIs, and one patient had progressed following more than three lines of therapy. Before VIA treatment, five of eight patients (62.5%) had localized inoperable lesions (local tumor thrombosis and lymphatic metastasis) and three of eight (37.5%) had metastatic lesions of the lung, bone, or liver. The presence of SMARCB1/INI1 protein expression and gene deletion occurred in seven of eight (87.5%) cases evaluated. Seven of these patients were classified as classic-type ES and one was deemed proximal-type ES [1].

**Table 1:** Patients demographics

Patient characteristics	*N*=8	%
Gender		
Male	3	37.5
Female	5	62.5
Age (Median, IQR) years	36.0 (10.2, 61.8)	
Location of primary lesion		
Trunk	0	0.0
Upper extremities	4	50.0
Lower extremities	3	37.5
Other sites	1	12.5
INI 1 loss		
Yes	7	87.5
No	0	0.0
Not known	1	12.5
ECOG score		
0–2	7	87.5
> 2	1	12.5
Stage at initial diagnosis		
Localized (including local tumor thrombosis and lymphatic metastasis)	5	62.5
Metastatic	3	37.5
Location of metastatic lesions		
Lung	0	0.0
Lymph nodes	3	60.0
Multiple organ metastasis	2	40.0
Lines of previous systemic therapy		
0 line	2	25.0
1 line	1	12.5
2 lines	4	50.0
3 lines and more	1	12.5
Best overall response		
PR	3	37.5
SD	5	62.5
PD	0	0.0
PFS (Mean, 95%CI) months	8,2 (8.0, 8.3)	
OS (Mean, 95%CI) months	74.8 (54.1, 95.4)

**Table 2:** Detailed information and treatment courses for each patient

Patient no.	Location of primary lesion	Previous systemic treatment	Locations of lesions for evaluation	Duration of using current treatment (m)	The reason for treatment cease	Best overall response	Status for last follow up	Overall survival (m)
1	Left forearm	None	Primary lesion and lymphatic metastasis	6.5	Patient’s intention	SD	AWD	83.7
2	Left thigh	AI chemo, Anlotinib	Relapsed thigh lesion, vein tumor thrombosis, Subcutaneous metastasis	2.9	Adverse events	PR	AWD	86.3
3	Right humerus	None	Multiple bone metastasis and tiny pulmonary metastasis	3.6	Adverse events	SD	AWD	9.5
4	Right finger	AI chemo, albumin paclitaxel	Primary relapsed lesion and lymphatic metastasis to axillary fossa	7.2	No stopping treatment	PR	AWD	14.7
5	Right inguinal fold	AI chemo, apatinib, anlotinib, cabozantinib, pazopanib, lenvatinib	Multiple bone metastasis and retroperitoneal lymph node metastasis	8.3	Adverse events (tumor rupture in her retroperitoneum)	PR	AWD	69.4
6	Left upper arm	AI chemo, apatinib	Primary lesion and lymphatic metastasis	8.0	Definitive surgery (amputation)	SD	NED	23.8
7	Left inguinal fold	AI chemo+anlotinib	Primary relapsed lesion locally involving pelvis and lymphatic metastasis	3.0	Adverse events (diarrhea)	SD	DOD	17.2
8	Left upper arm	AI chemo, anlotinib	Primary lesion and lymphatic metastasis, multiple pulmonary metastasis	10.0	COVID-19	SD	AWD	22.8

*Abbreviations*: *SD* Stable disease, *PR* Partial response, *AWD* Alive with disease, *AI* Chemo adriamycin and ifosfamide chemotherapy, *NED* None evidence of disease, *DOD* Died of disease, *COVID-19* Coronavirus disease 2019, *m* Month

### Efficacy

**Fig. 1:**
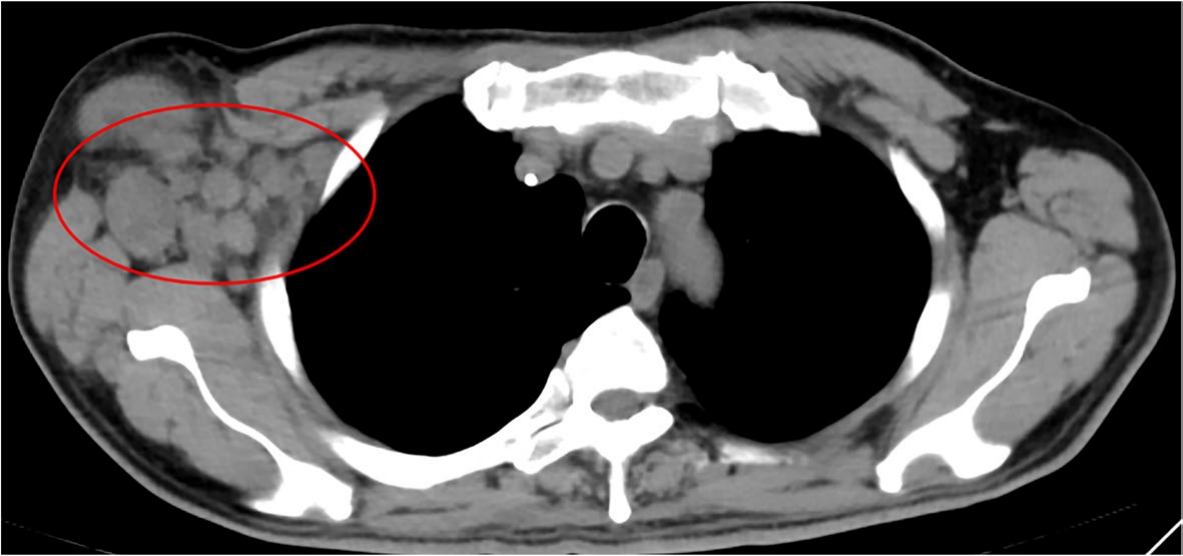
An epithelioid sarcoma patient with primary lesion located at right finger (patient number 4 in table 2) developed lymphatic metastasis to right axillary fossa before the combination therapy of irinotecan, vincristine and anlotinib

**Fig. 2:**
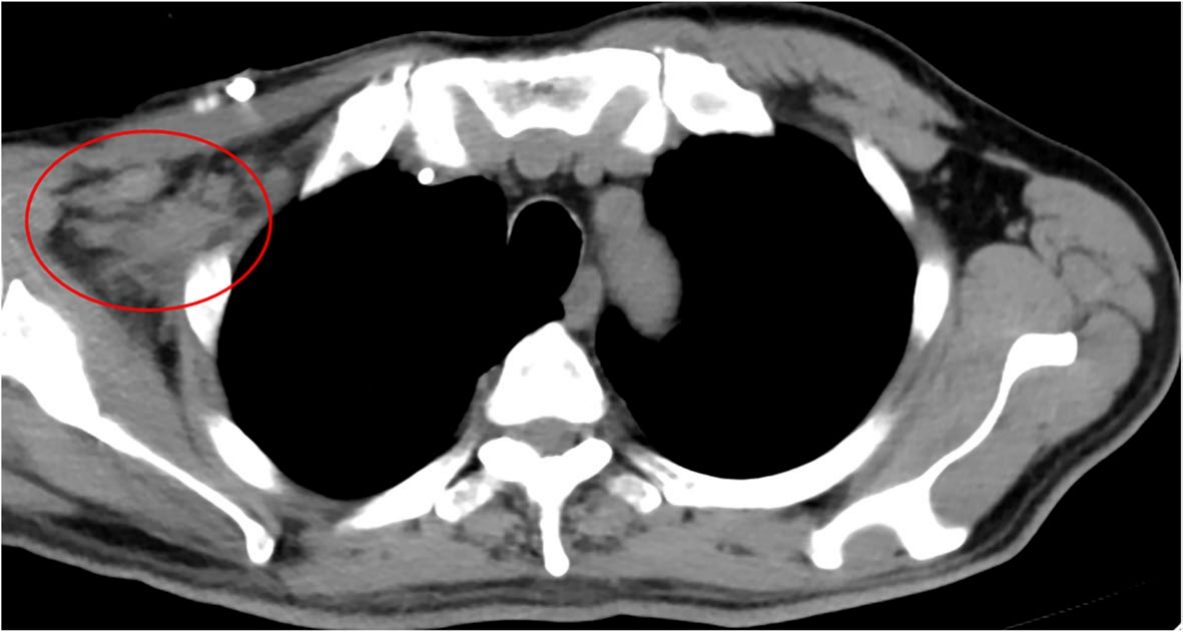
Partial Response was noticed shortly after 3 cycles of this combination therapy in the right axillary fossa lymph node metastasis (patient number 4 in Table 2)

**Fig. 3:**
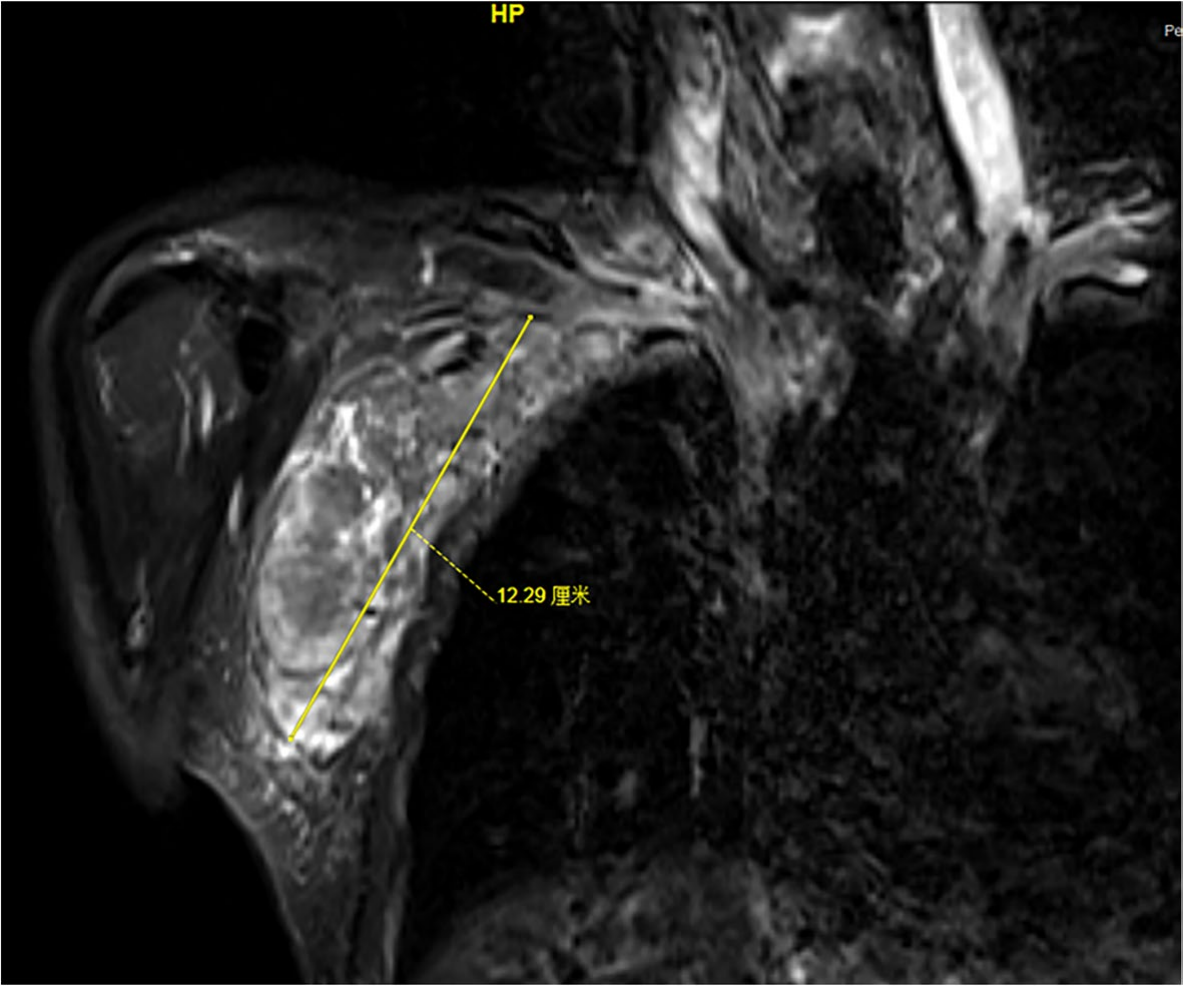
Manifestation of coronal scan of magnetic resonance imaging for the patient with right axillary fossa lymphatic metastasis before this combination treatment, who has progressed upon two lines of chemotherapy

**Fig. 4:**
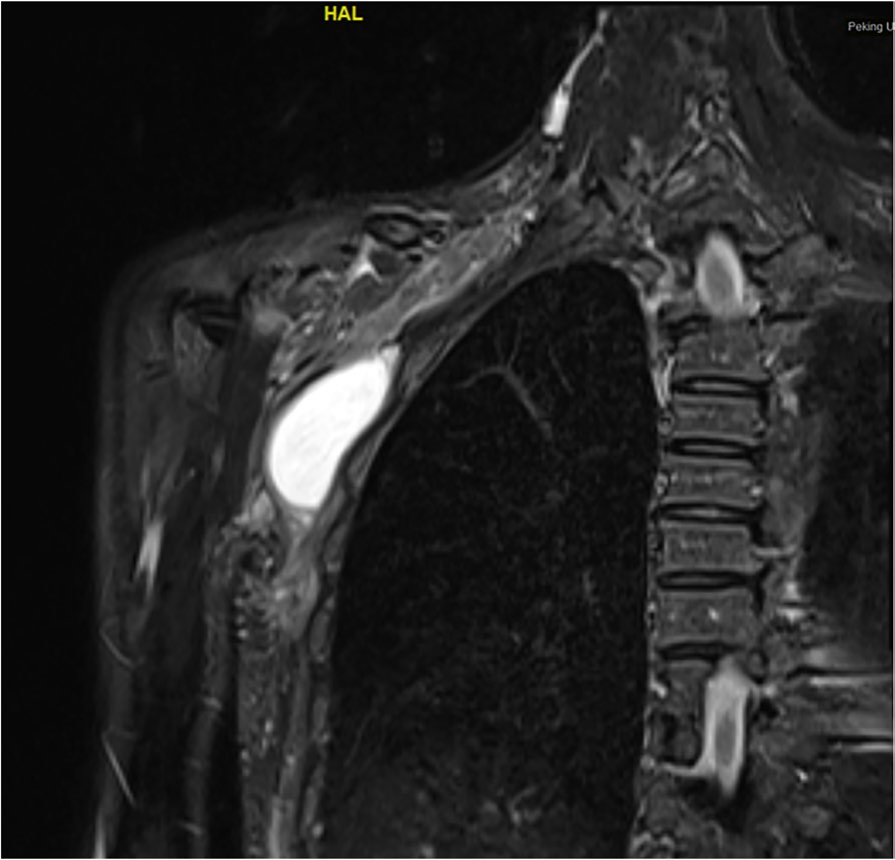
The magnetic resonance imaging manifestation after 3 cycles of irinotecan, vincristine and anlotinib, which induced shrink of the tumor as well as liquefactive necrosis

**Fig. 5:**
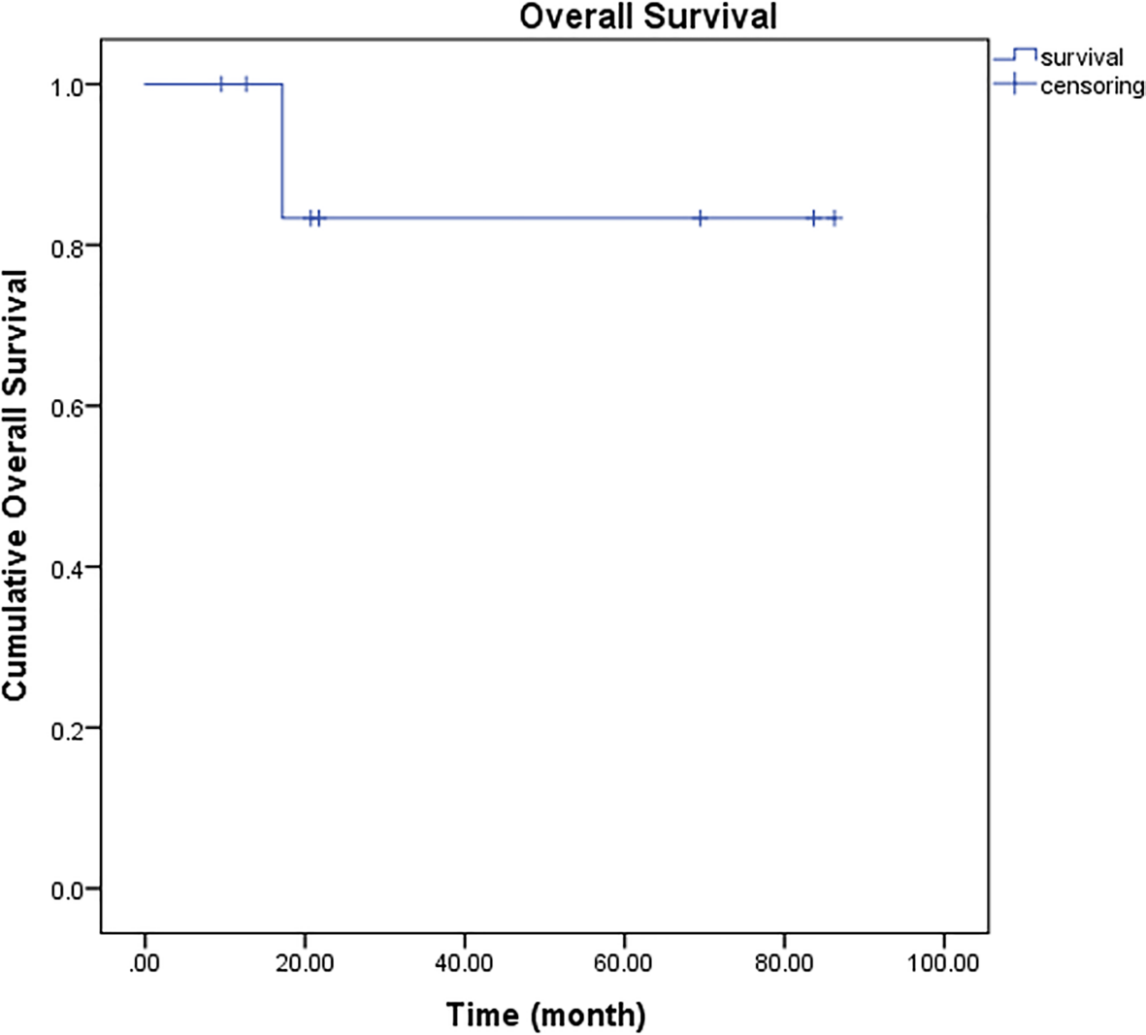
The Kaplan-meier estimate survival curve for overall survival in this group of patients from diagnosis to death

Based on RECIST1.1 criteria, three of eight (37.5%) patients experienced a partial response (PR) while five of eight (62.5%) had stable disease (SD) (Figs. 1 and 2) [23]. The DOR was 2.9, 7.2, and 8.3 months in the three patients, with two patients stopping treatment due to adverse events (AEs). The one patient is currently using the combination therapy for clinical benefit and is tolerating it well (Figs. 3 and 4). None of the patients experienced Progression of Disease (PD) or had ever progressed on this treatment combination. All of the patients who were disease stable ceased treatment due to reasons other than progression. Due to many difficulties (AEs, patient’s intention, local therapy, and COVID-19 pandemic control) and the small sample size in this case series, PFS could not be calculated by the Kaplan–Meier Method. However, with a follow-up time ranging from 9.5 to 86.3 months, we were able to determine that the median Overall Survival (OS) was 21.3 (95% CI, 54.1, 95.4) months and the 5-year OS rate was 83.3% (standard error 15.2%) (Fig. 5).

### Toxicity

**Fig. 6:**
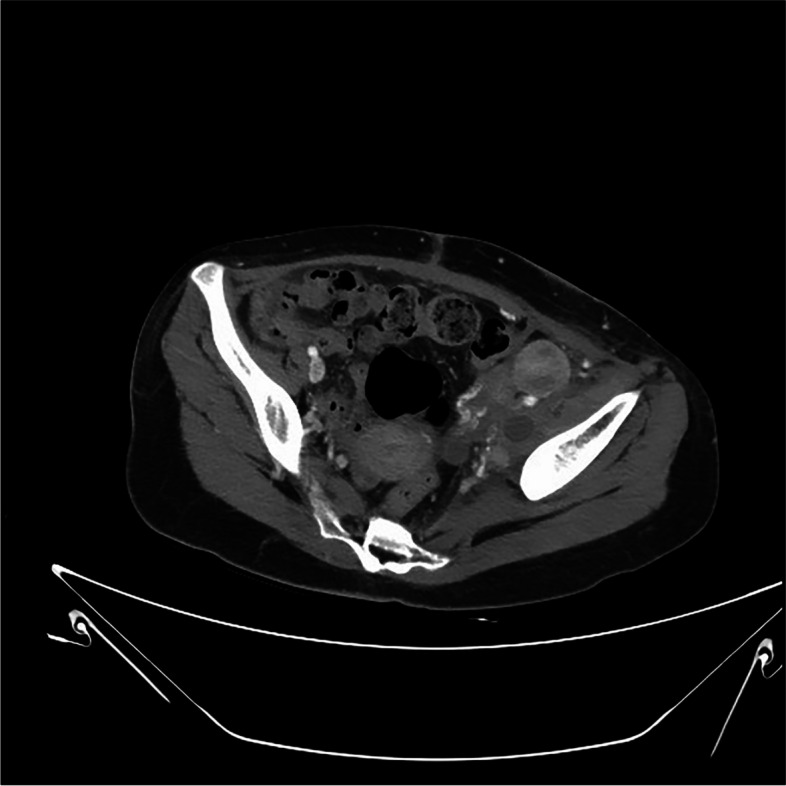
A female patients (patient number 5 in Table 2) with epithelioid sarcoma in right inguinal fold experienced tumor rupture in her retroperitoneum. This was her computerized tomography (CT) scan before rupture

**Fig. 7:**
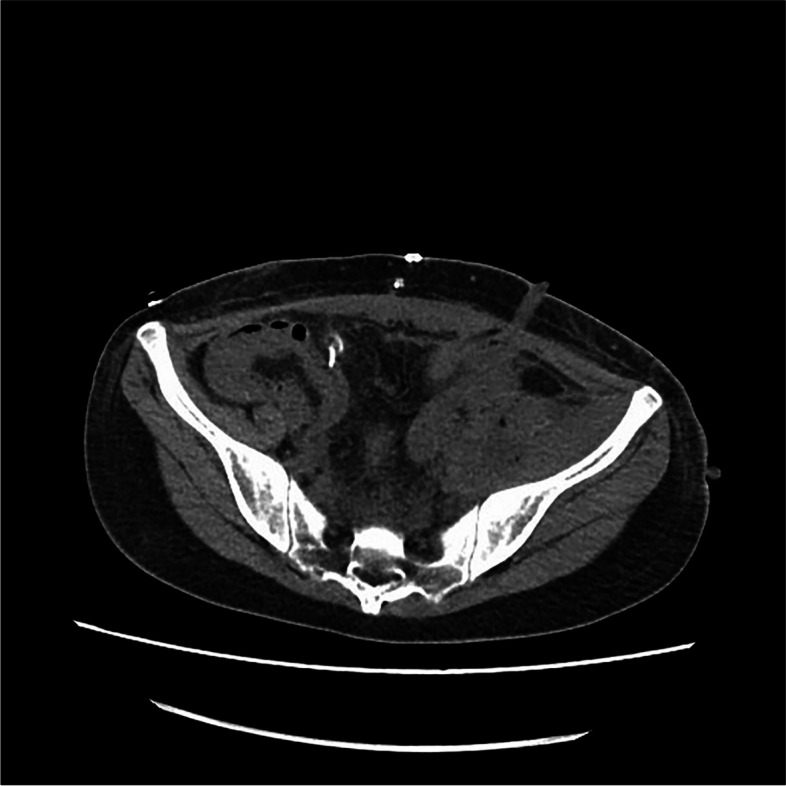
The CT scan taken shortly after her tumor rupture in her retroperitoneum with drainage tube after Emergent debridement surgery (patient number 5 in Table 2). Following debridement and suturing, this female patient recovered from the infection and continued using single chemotherapy of irinotecan

All of the AEs related to the therapy were routinely recorded in the electronic medical record using Common Terminology Criteria for Adverse Events (CTCAE) (version 5.0) [26]. Grade 3–4 AEs are summarized in Table 3. The most common Grade 3 or 4 AEs included myelosuppression (92.6%) (particularly neutropenia and anemia), diarrhea (81.5%), and nausea and vomiting (68.6%). Notably, one patient experienced pelvic soft tissue necrosis and rupture (partially tumor-related and partially treatment-related), which required surgery for drainage (Figs. 6 and 7). Following debridement and suturing, this female patient recovered from the infection and continued using single chemotherapy treatment with irinotecan (patient number 5 in Table 2). However, bladder perforation occurred shortly after this due to previous radiation and tumor rupture, which required a second surgery for debridement and uretero-cutaneous diversion. One patient with a large left inguinal fold mass (patient 7 in Table 2) had grade 4 diarrhea and dehydration leading to loss of consciousness and was admitted directly to the intensive care unit (ICU) for palliative treatment. This suggests that the combination treatment should be accompanied with appropriate preventive measures to avoid severe toxicities.

**Table 3:** Grade 3 and 4 Toxicities observed in 8 patients (54 courses) according to CTCAE 5.0

Toxicity	Grade 3		Grade 4	
	Events (%)	Patients (%)	Events (%)	Patients (%)
Blood and lymphatic system disorders				
Neutropenia	44 (81.5)	8 (100.0)	6 (11.1)	2 (25.0)
Platelet count decreased	30 (55.6)	7 (87.5)	4 (7.4)	2 (25.0)
Anemia	35 (64.8)	6 (75.0)	2 (3.7)	2 (25.0)
Febrile neutropenia	8 (14.8)	6 (75.0)	1 (1.9)	1 (12.5)
Gastrointestinal disorders				
Diarrhea	40 (74.1)	8 (100.0)	4 (7.4)	2 (25.0)
Nausea and vomiting	36 (66.7)	8 (100.0)	1 (1.9)	1 (12.5)
Mucositis oral	5 (9.3)	5 (62.5)	0 (0.0)	0 (0.0)
Infections and infestations				
Abdominal infection	4 (7.4)	2 (25.0)	1 (1.9)	1 (12.5)
Metabolism and nutrition disorders				
Hypokalemia	21 (38.9)	8 (100.0)	2 (3.7)	2 (25.0)
Anorexia	14 (25.9)	8 (100.0)	1 (1.9)	1 (12.5)
Dehydration	11 (20.4)	3 (37.5)	1 (1.9)	1 (12.5)
Weight loss	12 (22.2)	3 (37.5)	0 (0.0)	0 (0.0)
Injury, poisoning and procedural complications				
Pelvic soft tissue necrosis	0 (0.0)	0 (0.0)	1 (1.9)	1 (12.5)
Bladder perforation	0 (0.0)	0 (0.0)	1 (1.9)	1 (12.5)

## Discussion

The present study, which had an ORR of 37.5%, provides evidence for the use of an alternative systemic treatment option for refractory ES. In indolent, locally aggressive, ultra-rare soft-tissue sarcomas, systemic treatment focused on ORR may effectively reduce tumor load. This, in turn, may convert inoperable ES to an operable state and allow late focal stage patients to avoid drug therapy and remain disease-free. The rationale for the combination of irinotecan, vinsristine, and Anlotinib in the present study was based on the theory that the addition of chemotherapy to TKIs may overcome the cytostatic properties of molecular targeted agents, especially with regard to anti-angiogenesis drugs [27]. The success of the LEADER study [28], studies reporting Lenvatinib used with etoposide plus ifosfamide in osteosarcoma [27], and other similar combinations [29] led us to test other cytotoxic agents commonly used in pediatric sarcoma patients in combination with Anlotinib. Anlotinib was used because it was deemed to be less toxic in a previous trial and is therefore more suitable for combination therapy [30, 31]. Tazemetostat was not used in these advanced patients because the agent had not yet been approved for application in mainland China when the patients were treated (2015–2021). There were no better systemic options for ES patients that had progressed on anthracycline-based chemotherapy regimens or TKIs.

Based on the results of a nonrandomized Phase II study (EZH-202; ClinicalTrials.gov: NCT02601950) that included INI1 negative advanced ES, the US FDA granted accelerated approval for tazemetostat (Tazverik R; Epizyme, Inc., 400 Technology Square, MA, USA) for the treatment of adult advanced ES in January 2020. This is a first-in-class, oral, small-molecule selective inhibitor of EZH2 and was also the first epigenetic regulating agent approved for soft tissue sarcoma [3]. Studies of epigenetic agents have become an important research focus as this is another tumor regulatory mechanism in tumor pathogenesis. However, the efficacy of EZH2 was subtle in clinical trials of its use in other sarcomas, especially when used as a single-agent therapy [32, 33]. In ES, we found an ORR of 15% in the modified intention-to-treat analysis, with durable responses and a median progression-free survival of 5.5 months [3]. In addition, most treatment-induced AEs were mild and tolerable [27], which suggests that this drug might be more suitable for maintenance in high-risk ES. It should be noted that a report by SL Spunt, N Francotte, GL De Salvo, Y-Y Chi, I Zanetti, A Hayes–Jordan, SC Kao, D Orbach, B Brennan, AR Weiss, et al. [2] showed that 71.3% of ES patients who experienced full resection of all tumor lesions achieved 5-year event-free survival (95% CI, 56.7.0–81.7). Thus, ORR is important in ES as it may allow patients to undergo wide resection or radiotherapy. We have summarized the most promising, recent systemic treatments for ES in Table 4. We noted that chemotherapies such as doxorubicin, ifosfamide, gemcitabine, docetaxel or these agents in combination, can induce greater ORR and should be considered more in neoadjuvant settings.

**Table 4:** Summary of systemic studies in epithelioid sarcoma

Author	Institutions	Year	No.	Systemic treatment	ORR (%)	mPFS (month)
Sheri L. Spunt et al.	COG; EpSSG	2005–2015	63	Mainly based on doxorubicin and ifosfamide chemotherapy	50%	5.4 for the metastatic high-risk series
Dario Baratti et al.	Fondazione IRCCS-INT	1986–2005	34	A combination of Adriamycin and ifosfamide	NA	NA
Monika Sparber-Sauer et al.	CWS trials	1981–2016	67	VAIA or CEVAIE;	35%	NA
				Gemcitabine and Docetaxel	33%	
Scott M. Schuetze et al.	SARC009	2007–2016	7	Dasatinib	28.6%	7.9
Mrinal Gounder et al.	32 hospitals and clinics	2015–2017	62	Tazemetostat	15%	5.5
Current Study	PKUPH	2017–2021	8	Irinotecan, vincristine and TKIs	37.5%	8.0

*Abbreviations*: *No.* Patient Number, *ORR* Objective response rate, *mPFS* Median progression-free survival, *COG* Children’s Oncology Group, *EpSSG* European paediatric soft tissue Sarcoma Study Group, *INT* Istituto Nazionale Tumori, *CWS* Cooperative Weichteilsarkom Studiengruppe, *SARC009* Sarcoma Alliance Through Research and Collaboration, *CBR* Clinical benefit rate, *NA* Data not available, *VAIA* Vincristine, dactinomycin, ifosfamide and alkylating agent, *CEVAIE* Carboplatin, etoposide, vincristine, Adriamycin and ifosfamide, *PKUPH* Peking University People’s Hospital, *TKIs* Tyrosine kinase inhibitors

Nevertheless, this study investigated an alternative systemic treatment option with a predilection to more toxic profiles compared to previous studies [3, 13]. Diarrhea, nausea and vomiting, and myelosuppression were found to be severe. Therefore, prophylactic therapy should always be administrated to avoid these unexpected consequences [20]. Even when full tumor removal is achieved, physicians should remain vigilant for tumor rupture or visceral perforation, partly due to the effects of TKIs [34, 35]. In addition, single chemotherapy with irinotecan should be considered later in clinical practice to assess its efficacy and toxicity.

This study had several limitations. First, this was a retrospective study with a small sample size, making statistical calculations difficult. Thus, we could only present each case in detail in Table 2 to demonstrate the results. Second, most patients ceased the treatment for various reasons other than progression, leading to a lack of PFS data. Thus, PFS was not investigated in this study. As a result, we focused on the VIA regimen and its ORR, which is not adequate to address the activity of the regimen. Third, this cohort of patients was generally heavily treated with various modalities including surgery, radiation, and other biological agents, making interpretation of the data difficult. To guarantee the uniformity of the data, the inclusion criteria were retrospectively designed and rigidly implemented, leading to the small sample size.

## Conclusions

We explored a novel treatment regimen for ES patients (a combination of irinotecan, vincristine, and Anlotinib) used in our past clinical practice. This treatment regimen resulted in relatively high response rates. Special attention should be paid to its toxicities, which were in accordance and comparable with similar combinations. Further investigation using prospective trials should be carried out to complement these findings.

## Data Availability

The datasets used and/or analysed during the current study available from the corresponding author on reasonable request.

## Abbreviations

AEs: Adverse events
AJCC: American Joint Committee on Cancer
COVID-19: coronavirus disease 2019
CTCAE: Common Terminology Criteria for Adverse Events
DOR: Duration of response
ECOG: Eastern Cooperative Oncology Group
ES: Epithelioid sarcoma
FDG-PET: [^18^F]2-fluoro-2-deoxy-D-glucose-positron emission tomography
ICU: Intensive care unit
MRI: Magnetic resonance imaging
NGS: Next-generation sequencing
ORR: Objective response rate
OS: Overall survival
PD: Progression of Disease
PFS: Progression-free survival
PR: Partial response
SD: Stable disease
TKI: Tyrosine kinase inhibitor
VIA: Vincristine, and Anlotinib
